# Does Digital Ad Exposure Influence Information-Seeking Behavior Online? Evidence From the 2012 Tips From Former Smokers National Tobacco Prevention Campaign

**DOI:** 10.2196/jmir.4299

**Published:** 2016-03-16

**Authors:** Annice Kim, Heather Hansen, Jennifer Duke, Kevin Davis, Robert Alexander, Amy Rowland, Jane Mitchko

**Affiliations:** ^1^ RTI International Research Triangle Park, NC United States; ^2^ Centers for Disease Control and Prevention Office on Smoking and Health Atlanta, GA United States

**Keywords:** tobacco cessation, health, Internet, monitoring and evaluation

## Abstract

**Background:**

Measuring the impact of online health campaigns is challenging. Ad click-through rates are traditionally used to measure campaign reach, but few Internet users ever click on ads. Alternatively, self-reported exposure to digital ads would be prone to recall bias. Furthermore, there may be latency effects whereby people do not click on ads when exposed but visit the promoted website or conduct campaign-related searches later. Online panels that unobtrusively collect panelists’ Web behavior data and link ad exposure to website visits and searches can more reliably assess the impact of digital ad exposure. From March to June 2012, the Centers for Disease Control and Prevention aired the national Tips From Former Smokers (Tips 2012) media campaign designed to encourage current smokers to quit. Advertisements ran across media channels, and the digital ads directed users to the Tips 2012 campaign website.

**Objective:**

Our aim was to examine whether exposure to Tips 2012 digital ads influenced information-seeking behaviors online.

**Methods:**

ComScore mined its panelists’ Web behavior data for unique codes that would indicate exposure to Tips 2012 ads, regardless of whether panelists clicked the ad or not. A total of 15,319 US adults were identified as having been exposed to a Tips 2012 campaign ad. An equal number of unexposed adults (N=15,319) were identified and matched on demographics and Internet use behavior to the exposed group. Panelists’ Web behavior data were mined for up to 4 weeks after initial Tips 2012 ad exposure to determine whether they visited the Tips 2012 campaign website or other cessation-related websites (eg, nicotine replacement therapy site) or conducted searches for campaign-related topics (eg, quit smoking).

**Results:**

The proportion of exposed adults visiting the Tips 2012 sites increased from 0.4% in Week 1 to 0.9% 4 weeks after ad exposure, and these rates were significantly higher than in the unexposed group (0.1% in Week 1 to 0.4% in Week 4, *P*<.001) across all weeks examined. The proportion of exposed panelists visiting other cessation websites increased from 0.2% in Week 1 to 0.3% 4 weeks after initial ad exposure, and these rates were significantly higher than in the unexposed group (0.0% in Week 1 to 0.2% in Week 4, *P*=.001 to *P*=.019) across all weeks examined. There were no significant differences in searches for campaign-related topics between the exposed and unexposed group during most of the weeks examined.

**Conclusions:**

These results suggest that online ad exposure is associated with confirmed visits to the Tips 2012 campaign sites and visits to other cessation websites and that these information-seeking behaviors occur up to several weeks after ad exposure. Web behavior data from online panels are useful for examining exposure and behavioral responses to digital campaign ads.

## Introduction

Smoking is the leading cause of preventable deaths in the United States, accounting for approximately 480,000 deaths annually [1]. An extensive body of research demonstrates that mass media campaigns are an effective strategy to encourage smoking cessation that contributes to reductions in adult smoking prevalence rates [2-4]. Historically, campaigns have placed advertisements on traditional broadcast media, such as television and radio, to inform target audiences about the dangers of tobacco use and to encourage use of cessation resources, such as a telephone quitline. Increasingly, campaigns have added digital advertising to reach audiences online and to drive visits to the campaign websites with cessation resources. While much is known about the impact of television ads on cessation-seeking behaviors with best practice recommendations to guide media strategy and planning of television and radio campaigns [5], very little is known about the impact of digital ads on health information-seeking behaviors online.

In 2012, the Centers for Disease Control and Prevention (CDC) launched Tips 2012 From Former Smokers (Tips 2012)—the first federally funded, national tobacco paid-media education campaign. The Tips 2012 campaign advertisements aired nationally from March to June 2012 on cable television, radio, online, print, and outdoor media (eg, billboards). Campaign ads featured former smokers sharing their stories about the daily challenges of living with smoking-related illnesses. To provide smokers with resources and information about quitting, Tips 2012 television ads promoted the 1-800-QUIT-NOW telephone quitline portal and the National Cancer Institute’s (NCI) Smokefree website [6]. The digital campaign consisted of display, video, mobile, and search ads that were intended to reach online audiences and to direct them to the Tips 2012 website [7]. The campaign also disseminated cessation information to audiences via CDC’s Tobacco Free Facebook page [8] and Twitter handle [9] and CDC’s StreamingHealth YouTube channel [10]. The Tips 2012 campaign was effective in changing tobacco-related knowledge, beliefs, and intentions to quit smoking [11]. Further, it influenced an estimated 1.64 million smokers to make a quit attempt and 100,000 smokers to remain abstinent permanently [12]. Additionally, calls to the quitline increased by 132%, and the number of unique visitors to the Smokefree.gov cessation website increased 428% during the campaign [13].

The purpose of this study is to examine the impact of the Tips 2012 digital advertisements on cessation information-seeking behaviors online. Traditionally, the impact of campaign ad exposure on short-term tobacco-related outcomes like information-seeking behaviors and awareness of campaign messages has been examined by linking gross rating points (ie, reach x frequency of ad exposure) as an exogenous observational measure of television ad exposure to survey responses (eg, [3,14-16]). However, self-reported survey responses may not accurately measure the potential impact of digital ad exposure on information-seeking behavior online because they rely on participant recall of the promoted website and campaign-related topics that may be prone to recall bias.

Measuring the impact of digital campaign advertisements is challenging. Ad impressions and click-through rates (CTRs) are traditionally used to measure message reach, but they are limited because only a small fraction of Internet users ever click on ads [17], and CTRs are not linked to behavioral outcome data, such as online information-seeking behavior. Even if ads are not clicked, incidental ad exposure can affect brand/campaign awareness [18,19]. Furthermore, there may be latency effects whereby people do not click on ads at the time of exposure but visit the promoted website or conduct searches on campaign-related topics later [17]. Website analytics programs (eg, Google Analytics, Adobe SiteCatalyst) provide some insights into the impact of online ads (eg, what proportion of traffic originated from clicks on paid search ads), but not for all ad types (eg, video paid ads) and it does not link online ad exposure to website visits. An alternative approach would be to assess self-reported exposure to digital ads via surveys. However, this approach would especially be prone to recall bias given the extensive diversity of websites users may visit on any given day, the different types of ads (eg, display, search, video, social), and the manner in which users may access the Internet (via computer vs mobile devices) that may influence the number and type of ads delivered and viewed.

A more accurate approach to measuring digital ad exposure and behavioral impact is needed. A panel-based method that unobtrusively collects Web behavior data and can link ad exposure to online information-seeking behaviors at the individual level may be a more reliable method for measuring online campaign effects [20]. Several companies (eg, comScore [21], Nielsen [22]) have Web-based panels in which members agree to install a software on their computers that unobtrusively captures data about their online behavior, including websites visited, searches conducted, and whether a specific ad was delivered on a site they visited, regardless of whether they clicked on the ad or not. In this study, we used comScore’s Web panel of approximately 1 million US adult Internet users to identify those who were exposed to the campaign and to assess whether exposure to the Tips 2012 digital ads was associated with (1) visits to the Tips 2012 campaign website, the Smokefree.gov cessation site, and other non-campaign-related cessation sites and (2) searches for campaign-related topics and general information about cessation.

## Methods

### Tips 2012 Digital Advertising

**Figure 1:**
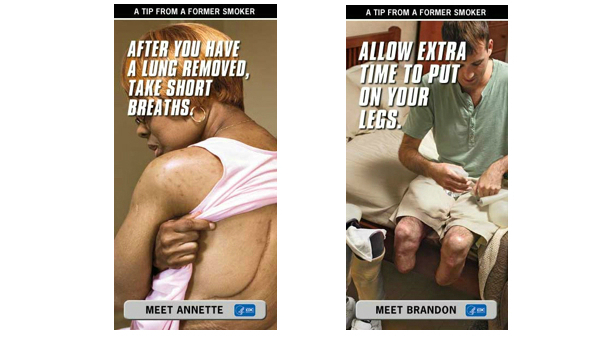
Provides examples of CDC Tips 2012 campaign-related advertisements.

The digital campaign consisted of display, video, mobile, and search ads that were intended to reach the target audience of adult smokers aged 18-54 and to direct them to the Tips 2012 website. All digital ads ran from March 19-June 10, 2012. Display ads were animated or static and appeared at the top or sidebar of popular websites, such as weather.com, to attract target audiences. The display ads were placed on select websites and ad networks and highlighted the stories of former smokers Annette, Brandon, Shawn, Roosevelt, Suzy, and Terrie (see examples in Figure 1). The campaign also featured a cessation support ad of formers smokers who had quit (“Cessation”) and the “Asthma” ad, which highlighted the harmful effects of exposure to secondhand smoke. Display ads were tagged with the CDC Tips 2012 website so that viewers who clicked on the ad were directed to the Tips 2012 site. Approximately 372 million impressions of digital ads were served, and they generated 489,000 clicks for a CTR of 0.13%, which exceeds the industry standard of 0.08% for display ad CTRs [23].

Video ads are generally shown before or after other video content, such as an online television show or music video. Video ads ran on 29 websites and featured the same 30-second ads that ran on television. Video ads were tagged with the CDC Tips 2012 website so that viewers who clicked on the ad were directed to the Tips 2012 site. For video ads, approximately 407 million impressions were served, and these ads generated 4.2 million clicks for a CTR of 1.05%, which exceeded industry standards of 1.03% for video ad CTRs [24].

Search ads appear at the top and sidebar of search results so that when consumers type in any of the paid search terms (eg, how to quit smoking), the top result retrieved is the Tips 2012 site. Tips 2012 search ads were purchased for the two top search engines, Google and Yahoo. For search ads, 22.6 million impressions were delivered via search ads, and these ads generated 224,811 clicks for a CTR of 1.0%, which was equal to the industry standard of 1.0% [25] for search ad CTRs.

### Panel Data

The data for this analysis came from comScore, a market research company that unobtrusively collects Web behavior data on 1+ million US Internet users to measure trends in consumer behaviors online. Panelists download tracking software on their computers that enables comScore to track their Web behavior, including every website they visit, searches they conduct, purchases they make, and ads that are delivered on sites visited, regardless of whether the ads are clicked or not. These data are then aggregated and weighted to provide national estimates on consumer behaviors online. The panel is a convenience sample with panelists largely recruited via nonprobability-based sampling methods (eg, online ads, partner websites). However, a subsample is recruited via random-digit-dialing to calibrate the post-stratification weights that comScore uses to project its estimates to the US Internet population. Panelists are provided incentives for participation such as free online games and charitable donations on panelists’ behalf.

## Measures

### Exposure to Tips 2012 Digital Display and Search Ads

The Tips 2012 campaign’s media contractor provided the “tags” (hash identification code, hypertext markup language source code) to each of the Tips 2012 digital display ads. Using this information, comScore mined its panelists’ Web behavior data for these display ad tags and exposure to sponsored links (search ads) to identify individuals who were exposed and not exposed to the Tips 2012 digital campaign from March 19-June 10, 2012. Video and mobile ad exposure was not examined in this study. Among comScore’s approximately 1 million US adults who were active panelists (ie, tracking software was installed and sending data) during the March 19-June 10, 2012 time period, 15,319 panelists were identified as having been “exposed” to the Tips 2012 digital display and/or search ads. For a control group, an equal number of “unexposed” adults (N=15,319) were matched to the exposed group on demographics and Internet use behavior (eg, time spent online) using propensity score matching nearest neighbor approach (Table 1). comScore uses propensity score matching to balance exposed and unexposed groups in order to isolate the effects of digital campaign ad exposure (eg, [17]). Propensity score matching has been widely used to adjust for selection bias in estimating campaign exposure effects (eg, [26]). Information about panelists’ smoking status was not available. Once the exposed and unexposed groups were identified, panelists’ Web behavior data, including websites visited and searches conducted, were mined for up to 4 weeks after initial Tips 2012 ad exposure.

**Table 1:** Demographic characteristics of exposed and unexposed panelists.

Demographic		Exposed panelists, % (N=15,319)	Unexposed panelists, % (N=15,319)
**Age**			
	18-24	15	11
	25-34	18	20
	35-44	20	21
	45-54	24	25
	55-64	14	15
	65+	9	8
**Race/Ethnicity**			
	White	46	50
	Black	22	20
	Asian	13	13
	Other	19	17
**Geography (United States)**			
	North East	20	19
	North Central	20	20
	South	37	40
	West	23	21
**Annual household income, USD**			
	Less than $25K	30	29
	$25K-50K	25	24
	$50K to <75K	21	23
	$75K to <100K	12	13
	More than $100K	12	10
Children in household		27	28

### Visit to Tips 2012 Campaign Websites

We examined whether panelists in the exposed and unexposed groups visited any of the campaign sites listed in Table 2, which includes the main Tips 2012 site and social media pages (CDC Tobacco Free Facebook page, CDC Tobacco Free Twitter handle, and CDC’s StreamingHealth YouTube channel) used to disseminate Tips 2012 messages. A visit was captured if the panelist clicked on the display ad (which sent them directly to the Tips 2012 website) or used other methods, such as clicking on search results, typing in the uniform resource locator (URL) directly into the browser, or clicking on a hyperlink from another site.

The Tips 2012 television ads directed audiences to NCI’s Smokefree.gov website because it offers extensive cessation resources. As a result, awareness of the Smokefree.gov website may be high and people may associate this site with the Tips 2012 campaign. Therefore, we also examined visits to NCI’s Smokefree.gov, its associated websites (Smokefree Women and Smokefree Espanol), and social media pages.

**Table 2:** Tips 2012 campaign and non-campaign websites.

Website			URL
**Campaign websites**			
	**CDC Tips 2012**		
		CDC Tips 2012 campaign website	cdc.gov/tobacco/campaign/Tips 2012/
		Tobacco Free Facebook page	facebook.com/cdctobaccofree
		Tobacco Free on Twitter (@CDCTobaccoFree)	twitter.com/CDCTobaccoFree/
		CDC StreamingHealth YouTube Channel	youtube.com/user/CDCStreamingHealth
	**NCI Smokefree**		
		NCI Smokefree website	Smokefree.gov
		Smokefree.gov on Twitter (@SmokefreeGov)	twitter.com/smokefreegov
		Smokefree Women website	women.smokefree.gov
		SmokefreeWomen onTwitter (@SmokefreeWomen)	twitter.com/SmokefreeWomen
		Smokefree Women YouTube Channel	youtube.com/SmokefreeWomen
		Smokefree Women Facebook page	facebook.com/smokefree.women
		Smokefree Espanol website	espanol.smokefree.gov
**Other non–campaign-related cessation sites**			
	**Cessation-related** ^a^		
		HealthWays cessation service	quitnet.com
		Alere cessation service	quitnow.net
		Legacy cessation service	becomeanex.org
		American Cancer Society cessation resources	cancer.org/Healthy/StayAwayfromTobacco/GuidetoQuittingSmoking/index
		American Lung Association cessation resources	lung.org/stop-smoking/
	**Nicotine Related Therapy (NRT)-related**		
		Nicoderm CQ patch	nicodermcq.com
		Nicotrol inhaler	nicotrol.com/
		Nicorette gum/lozenge/mini	nicorette.com
		Habitrol patch	habitrol.com
	**State cessation program websites** ^a^		
		Make Smoking History—Massachusetts	Makesmokinghistory.org
		Tobacco Free Florida	tobaccofreeflorida.com
		Tobacco Free Florida—Facebook	facebook.com/TobaccoFreeFlorida

^a^These are examples only, not the entire list. In total, 101 cessation sites were examined, including 10 national cessation-related sites, 4 NRT sites, and 87 state cessation program sites. This list of sites were compiled and reviewed by tobacco control researchers at Research Triangle Institute and CDC.

### Visit to Other Non–Campaign-Related Cessation Websites

We also examined visits to key national cessation sites (eg, [27]), state tobacco cessation sites (eg, [28]), and nicotine replacement therapy (NRT)-related sites (eg, [29]) (see Table 2). We examined panelists’ visits to these non-campaign-related cessation websites because websites with similar content may see increased visits through content-related searches by the exposed group when a campaign has low brand awareness. Additionally, seeing the Tips 2012 ad may trigger people’s recall of an existing state cessation program or NRT options they were intending to seek out.

### Search for Campaign-Related Topics

To determine whether exposure to Tips 2012 online ads influenced audiences to seek out additional information about the campaign, panelists’ search behavior data were mined for the occurrence of specific (eg, Tips 2012, Terri ad) and general (eg, quit smoking) campaign-related search queries on major search engines (eg, Google, Bing) as well as general websites with search functions (eg, YouTube). A list of 2270 potential search terms were examined based on top external keywords from Adobe SiteCatalyst for the CDC Tips 2012 website and Google Analytics for NCI’s Smokefree.gov site, as well as top keywords used in the digital ad campaign.

### Analysis

For each time period, we calculated the proportion of panelists in the exposed and unexposed groups who (1) visited the Tips 2012 campaign-related websites, (2) visited Smokefree-related websites, (3) visited other non–campaign-related cessation websites, and (4) conducted searches for any campaign-related key terms. Proportions were calculated separately for the exposed and unexposed groups and at each weekly time period (Week 1, Week 2, Week 3, and Week 4) after initial ad exposure. Results for Week 1 represent the proportion of exposed and unexposed groups who visited campaign sites or conducted searches within 1 week after first campaign ad exposure. Results for Week 2 represent the proportion of exposed and unexposed groups who visited campaign sites or conducted searches within Weeks 1 and 2 after first campaign ad exposure, and similarly Week 3 represent Weeks 1-3 after first campaign ad exposure, and Week 4 represent Weeks 1-4 after first campaign ad exposure. We conducted *t* tests to determine whether differences in proportions between the exposed and unexposed groups at each time period were statistically significant.

## Results

### Did Exposure to Tips 2012 Digital Ads Influence Visits to the Tips 2012 Campaign Sites and NCI Smokefree Sites?

**Figure 2:**
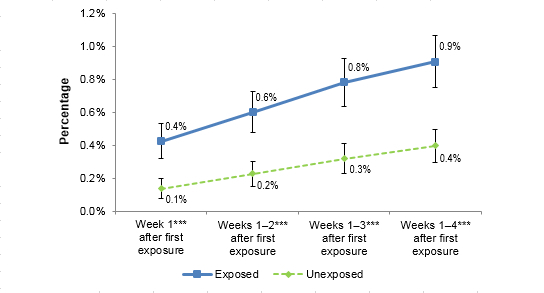
Shows the change in visits to CDC Tips 2012-related websites over the course of the campaign by digital ad exposure.

Figure 2 summarizes the proportion of exposed and unexposed panelists who visited any of the Tips 2012–related campaign sites. The proportion of exposed panelists visiting Tips 2012 sites increased from 0.4% in Week 1 to 0.9% in Week 4 after initial ad exposure. Unexposed panelists also visited Tips 2012 sites but at significantly lower rates from 0.1% in Week 1 to 0.4% at Week 4 (see Table 3). Significantly more panelists who were exposed to the Tips 2012 digital ads visited the campaign website compared to unexposed panelists at 1, 2, 3, and 4 weeks after initial ad exposure (*P*<.001). Very few panelists visited the Smokefree websites (˂0.1%), with no difference between the exposed and unexposed groups (data not shown).

**Table 3:** Visits to CDC Tips sites.

	Week 1	Weeks 1-2	Weeks 1-3	Weeks 1- 4
Exposed, % (95% CI)	0.4 (0.32-0.53)	0.6 (0.48-0.72)	0.8 (0.64-0.93)	0.9 (0.75-1.07)
Unexposed, % (95% CI)	0.1 (0.08-0.20)	0.2 (0.15-0.30)	0.3 (0.23-0.41)	0.4 (0.30-0.50)
*t* statistic	4.672	5.012	5.294	5.339
*P* value	<.001			

### Did Exposure to Tips 2012 Digital Ads Influence Visits to Other Cessation Websites?

**Figure 3:**
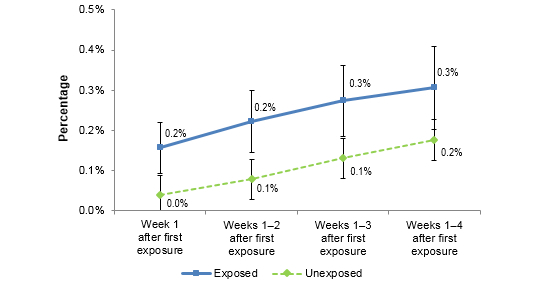
Shows the change in visits to cessation-related websites over the course of the campaign by digital ad exposure.

Figure 3 summarizes the proportion of exposed and unexposed panelists who visited any of the non–Tips 2012 cessation sites, including NRT sites, general cessation information sites, and state-specific cessation sites. The proportion of exposed panelists visiting other cessation websites increased from 0.2% in Week 1 to 0.3% in Week 4 after initial ad exposure. Unexposed panelists also visited other cessation websites but at lower rates, ranging from 0.0% in Week 1 to 0.2% at Week 4 (see Table 4). Significantly more panelists who were exposed to the Tips 2012 digital ads visited other cessation websites compared to unexposed panelists at 1, 2, 3, and 4 weeks after initial ad exposure (*P*=.001 to *P*=.019).

**Table 4:** Visits to other cessation sites (national, state, NRT).

	Week 1	Weeks 1-2	Weeks 1-3	Weeks 1- 4
Exposed, % (95% CI)	0.2 (0.09-0.22)	0.2 (0.14-0.30)	0.3 (0.19-0.36)	0.3 (0.20-0.41)
Unexposed, % (95% CI)	0.0 (0.00-0.08)	0.1 (0.03-0.13)	0.1 (0.07-0.20)	0.2 (0.10-0.26)
*t* statistic	3.135	3.053	2.560	1.973
*P* value	.001	.001	.005	.019

### Did Exposure to Tips 2012 Digital Ads Influence Searches for Campaign-Related Topics?

**Figure 4:**
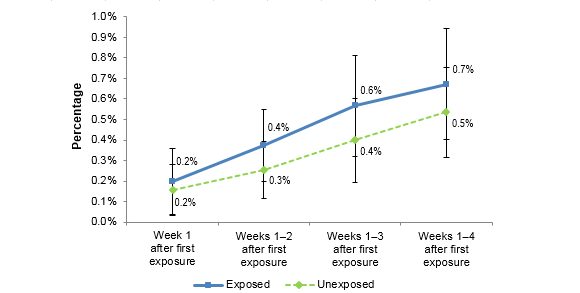
Shows the change in CDC Tips 2012 campaign-related searches over the course of the campaign by digital ad exposure.

Figure 4 summarizes the proportion of exposed and unexposed panelists who conducted searches on any of the campaign-related terms. The proportion of exposed panelists searching for cessation-related information increased from 0.2% in Week 1 to 0.7% in Week 4 after initial ad exposure. Unexposed panelists also conducted searches but at slightly lower rates from 0.2% in Week 1 to 0.5% at Week 4 (see Table 5). Rates of search behavior between exposed and unexposed panelists were only significantly different at 3 weeks after initial ad exposure (*P*=.032) and not at Weeks 1, 2, or 4.

**Table 5:** Search for any cessation terms.

	Week 1	Weeks 1-2	Weeks 1-3	Weeks 1- 4
Exposed, % (95% CI)	0.2 (0.04-0.36)	0.4 (0.20-0.55)	0.6 (0.32-0.81)	0.7 (0.40-0.94)
Unexposed, % (95% CI)	0.2 (0.03-0.28)	0.3 (0.12-0.39)	0.4 (0.19-0.60)	0.5 (0.32-0.75)
*t* statistic	0.377	1.033	1.037	0.773
*P* value	.414	.066	.032	.122

## Discussion

### Principal Findings

In summary, exposure to Tips 2012 digital display and search ads influenced visits to the Tips 2012 campaign-related websites, with visits occurring even up to 4 weeks after initial ad exposure. The total proportion of exposed panelists who visited the Tips 2012 website was higher than the total CTRs for digital display ads over the entire campaign period (0.9% vs 0.1%), suggesting that CTRs alone may underestimate campaign reach. These results are consistent with findings from a previous study that used a similar methodology to examine the influence of digital display ad exposure on visits to Florida state tobacco cessation website and social media pages [20]. Visits by comScore panelists to the Smokefree.gov website were low overall, with no differences by Tips 2012 digital ad exposure. This is likely due to the fact that Tips 2012 digital ads showed the Tips 2012 website URL and linked directly to the Tips 2012 website rather than Smokefree.gov, which was promoted only in broadcast television ads. Additionally, the Tips 2012 website was optimized in greater detail than the Smokefree.gov website to pick up traffic from searches (ie, search engine optimization), which may have also accounted for the fewer visits to Smokefree.gov relative to Tips 2012*.*

Interestingly, exposure to Tips 2012 digital ads influenced other information-seeking behavior online. The increased visits to other cessation sites (eg, NRT sites, quitnet) among those exposed to the Tips 2012 ads suggest that the campaign had an added benefit of driving traffic to other cessation sites. Therefore, state programs could leverage the timing of a national campaign like Tips 2012 and supplement it with additional local ad buys to drive target audiences to seek cessation resources. Those exposed to the Tips 2012 digital ads were not consistently more likely to search for campaign-related cessation information than those unexposed. This may be because both groups were exposed to messages from other channels (eg, Tips 2012 television/radio ads) that may have influenced similar patterns of information-seeking behavior online. In this study, we were not able to control for exposure to campaign messages from other media channels. However, since consumers are increasingly using multiple media devices simultaneously (eg, nearly 40% of Americans use their tablets or smartphones while watching television [26]), future studies should examine the relative influence of advertising exposure across media platforms on information-seeking behavior. Although digital advertising has historically been viewed as a competitor to television advertising, media companies like Nielsen increasingly believe that given the “reality of today’s consumers and their cross-platform habits, the two forms [of advertising] should be viewed as complementary rather than competing” ([30], p. 6). There may be cross-media platform effects, so future studies need to assess how television and digital ads can be optimized to achieve synergies in the intended behavioral outcome.

We saw an increase in the proportion of panelists visiting the Tips 2012 campaign site over time, but we cannot be sure whether this was due to a latency effect (ie, panelists remembering the campaign ad and visiting the site later) or increased exposure to multiple Tips 2012 ads during the post ad exposure 4-week follow-up period. In this study, we examined only first exposure to Tips 2012 digital display ad, but future studies should assess whether there is a dose-response relationship between the amount of digital advertising exposure and information-seeking behaviors online. Future studies should also examine the relative effectiveness of different types of digital ads (ie, display ads vs video ads vs mobile ads) on information-seeking behavior as we were able to examine exposure only to display and sponsored link search ads in this study.

While we found statistically significant differences in website visits and campaign-related searches between the exposed and unexposed groups, overall, the magnitude of the visits and searches and the difference between the groups were small. It is challenging to put these findings in context given the paucity of research on the effects of digital ad campaigns. Further research is needed to build the evidence base for digital media campaign effects.

### Strengths and Limitations

This study has several strengths. First, Web behavior data were collected unobtrusively, and ad exposure was measured regardless of whether the ad was clicked or not. Prior studies [32-36] have relied on self-reported survey data, the use of cookies, session identifiers, online ad campaign tracking (eg, Google AdWords), or website analytics tools (eg, Google Analytics), which have limitations such as recall bias or users deleting cookies that affect the accuracy of measuring campaign reach and exposure. Second, by matching unexposed and exposed groups on key demographics and online behavior, we were able to isolate the influence of digital ad exposure and minimize potential confounders. Third, we examined a comprehensive set of cessation websites (n=101) and search terms (n=2269) and were able to examine search behavior on major search engines like Google as well as any websites with search capabilities (eg, YouTube). To date, very few studies have examined how to measure online ad exposure and its effects on health information-seeking behavior online. To our knowledge, this is the first study that examines the impact of a national tobacco prevention campaign’s digital advertising strategy on information-seeking behaviors online. We chose to examine information-seeking behavior because studies have shown that it is associated with health knowledge and behavior choices [37,38]. However, we acknowledge that behavior change is a complex process and therefore information-seeking may not directly lead to health behavior change. Future studies should examine whether online information seeking influences behavior change by linking respondents’ Web visitation and search data to self-reported surveys.

This study also has limitations. First, we were unable to determine whether the increased visits to the campaign website were due to latency effects, increased level of digital ad exposure, or exposure to campaign content from other media channels. Future studies should examine the level and timing of ad exposure across media platforms to better understand dose-response relationships and cross-media effects. Second, we examined the influence only of display and search ad exposure, so we cannot determine whether these results would also translate to video or mobile ad exposure. Video ads may be more effective than display ads because advertisers can deliver more engaging and longer content in video formats and place these ads on sites like YouTube, which generate substantial traffic. Third, comScore’s panel is a convenience sample, and although estimates are weighted to the online population, results may not generalize to the US adult population. Fourth, we were unable to examine how smokers specifically responded to campaign ads because information on panelists’ smoking behavior was not available for this study. It is possible that panelists who were exposed to the digital ads were more likely to be smokers interested in quitting and therefore engaged in more information-seeking behavior online than the unexposed panelists. In this study, panelists were matched on demographic characteristics to isolate the influence of digital ad exposure, but future studies should investigate the impact of digital ad campaigns on specific subgroups. The audience that responds to digital ads is likely to be demographically and behaviorally different from the audience that responds to television ads. Therefore, understanding who is being reached can help campaign planners optimize media purchases across channels to reach target audiences most effectively. Finally, due to the confidential nature of proprietary data collected from comScore, we were unable to obtain detailed information about their methods (eg, specifics of data mining procedure) that may be needed to replicate studies of similar scope in the future. This is a common limitation when using data from digital analytics companies like comScore. For this reason, the national Media Rating Council conducts detailed audits of media industry companies to ensure that audience measurement services are valid, reliable, and ethical [39]. comScore’s methodology has been reviewed and accredited by the Media Rating Council [40]. We used comScore data because they are an industry leader in monitoring consumer online behavior, and collecting this type of passive Internet activity data from a large population based panel would have been cost prohibitive on our own. As we increasingly turn to data from digital analytics companies to understand online health behaviors, a broader discussion is warranted around the tradeoffs of using proprietary data with confidentiality restrictions and disclosing sufficient level of details needed to evaluate and replicate this research.

### Conclusions and Implications

The results of this study show that exposure to digital display and search ads is associated with confirmed visits to the campaign website up to several weeks after initial ad exposure regardless of whether the ad was clicked or not. Results also suggest that these ads may cue audiences to seek other cessation-related websites. Web behavior data from online panels are useful for examining exposure and behavioral responses to digital campaign ads because they provide a more comprehensive assessment of campaign impact than relying on ad impressions and CTRs alone. Future studies should examine the optimal dose needed to achieve information-seeking behaviors, the relative impact of different types of digital ads, cross-platform influences and synergies, and impact on specific subgroups like smokers. Digital advertising is a potentially powerful tool for motivating audience’s information seeking around behaviors that are targeted in campaign messages. Researchers and practitioners have an opportunity to harness the vast volume of digital data to provide a more evidence-based approach to designing and evaluating digital media campaigns and to help inform best practices.

## Abbreviations

CDC: Centers for Disease Control and Prevention
CTR: click-through rate
NCI: National Cancer Institute
NRT: nicotine replacement therapy
URL: Uniform Resource Locator
